# Powdery Mildew Caused by *Leveillula taurica* (Synonym: *Phyllactinia taurica*): A Global Challenge for Pepper Production

**DOI:** 10.1111/mpp.70128

**Published:** 2025-07-25

**Authors:** Anne Massire, Flavie Cussonneau, Sonia Elbelt, Carole Constant, Marc Bardin, Benoît Moury, Véronique Lefebvre

**Affiliations:** ^1^ INRAE, GAFL Montfavet France; ^2^ SAKATA Vegetables Europe SAS Uchaud France; ^3^ INRAE, Pathologie Végétale Montfavet France

**Keywords:** *Capsicum annuum*, disease management, *Erysiphaceae*, hemi‐endophyte, host genetic resistance, *Solanaceae*

## Abstract

**Background:**

Pepper powdery mildew, caused by the obligate fungal pathogen *Leveillula taurica* (asexual stage: *Oidiopsis taurica* (Lév.) Salmon 1906, synonym: *Oidiopsis sicula* Scalia 1902), poses a significant threat to pepper (*Capsicum* spp.) cultivation worldwide. This review delves into the taxonomy, geographical distribution, host range, disease symptoms, and life cycle of *L. taurica* and discusses strategies for managing its epidemics, with a focus on plant genetic immunity.

**Taxonomy:**

Phylum: *Ascomycota*; Class: *Leotiomycetes*; Order: *Helotiales*; Family: *Erysiphaceae;* Tribe: *Phyllactinieae;* Genus and species: *Leveillula taurica* (Lév.) Arnaud 1921. Synonym: *Erysiphe taurica* Léveillé 1851; in 2025, the species *Leveillula* taurica was renamed *Phyllactinia taurica*.

**Host Range and Distribution:**

*Leveillula taurica* exhibits a broad host range, infecting monocotyledonous and dicotyledonous plants of around 200 genera across 60 families, including both herbaceous plants and trees. It causes substantial agricultural losses, particularly in pepper crops. The pathogen is distributed globally, occurring on all continents except Antarctica.

**Disease Symptoms:**

Initial symptoms include chlorotic spots on the upper leaf surface, which may coalesce and turn necrotic over time. A white mycelial coating (conidia and conidiophores) appears on the lower leaf surface beneath these spots. Severe infections can lead to leaf curling, defoliation, sunburned fruits and reduced yield and quality. The disease is particularly destructive in greenhouses and regions with hot, dry days alternating with cool, humid nights.

**Disease Control:**

The hemi‐endophytic lifestyle of *L. taurica* complicates disease management. Effective management of *L. taurica* involves integrated strategies: regular crop monitoring for early detection, cultural practices to limit fungal development, biocontrol agents, and chemical treatments to prevent or eradicate infections, and the use of resistant plant varieties. Sulphur‐based fungicides, commonly used in organic farming, as well as demethylation inhibitors and quinone outside inhibitor (QoI) fungicides, have demonstrated efficacy; however, the emergence of QoI‐resistant isolates necessitates cautious use. Additionally, biocontrol agents, such as *Trichoderma* spp. and other mycoparasitic fungi, provide alternative tools by inhibiting fungal growth. Breeding and deploying resistant varieties provide a sustainable approach to managing this disease.

## Graphical abstract

This pathogen profile synthesises current knowledge on *Leveillula taurica* (synonym: *Phyllactinia taurica*) and genetic resistance in *Capsicum* species, covering taxonomy, life cycle, distribution, control strategies and future research directions.

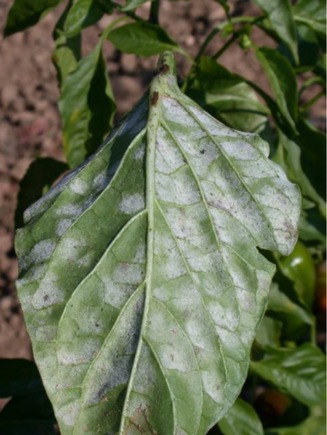

## 1 Introduction

Powdery mildew diseases, caused by fungi of the family *Erysiphaceae*, are prevalent worldwide and significantly impact agricultural production. The *Erysiphaceae*, historically classified under the order *Erysiphales* but now reassigned to the order *Helotiales* on the basis of recent phylogenetic studies, comprise over 900 species that infect more than 10,000 monocotyledonous and dicotyledonous plant species (Braun and Cook 2012; Dean et al. 2012; Glawe 2008; Vaghefi et al. 2022). These obligate biotrophic fungi primarily manifest as white powdery spots on leaves, stems and fruits that can enlarge and coalesce. However, symptom expression varies among fungal and plant species (Bardin and Gullino 2020).

For example, *Leveillula taurica* (Lév.) Arnaud 1921 (syn. *Phyllactinia taurica*) infection produces light‐green spots on the adaxial (upper) leaf surface, which progressively turn yellow. White sporulation of the fungus typically develops on the abaxial (lower) surface and occasionally on the adaxial side, potentially leading to necrosis. Infected pepper and tomato plants may exhibit necrotic lesions and extensive leaf shedding, significantly reducing yield (Palti 1988). Leaf shedding also exposes fruits to sunburn, further diminishing marketable fruit yield.

Powdery mildew diseases can cause severe economic losses across a wide range of crops. Without effective plant protection measures, yield reduction can reach up to 25% and 43% in peas and mung beans infected by *Erysiphe pisi* and *E. polygoni*, respectively (Bing et al. 2011; Vekariya et al. 2020), 50% in wheat caused by *Blumeria graminis* (Rana et al. 2022; Smith and Smith 1974), and 100% in grapevines infected by *E. necator* (Möth et al. 2023). In vegetables, *L. taurica* can reduce pepper yields up to 50% (Bademiyya and Ashtaputre 2019; Palti 1988) and devastate even entire greenhouses when early infections occur (Elad et al. 2007).

*Leveillula* *taurica* and its asexual stage *Oidiopsis taurica* (Lév.) Salmon 1906 affects a variety of crops, including peppers, tomatoes, eggplants, cucumbers, onions, fennels, legumes, sunflowers, cottons, and ornamental plants like nasturtiums and geraniums, as well as weeds and trees, making it a critical threat to global agriculture.

This review provides a comprehensive overview of current knowledge on *L. taurica* and the genetic basis of resistance to this pathogen in pepper (*Capsicum* spp.). We begin by exploring the taxonomic classification of *L. taurica*, highlighting its hemi‐endophytic life cycle. Next, we examine its geographical distribution and the various strategies available for disease control. We then review sources of resistance identified in pepper and the genetic determinism of resistance to *L. taurica*. Finally, we highlight knowledge gaps in the understanding of this pathosystem and discuss potential directions for future research.

## 2 Phylogenetics of the Genus *Leveillula*

The genus *Leveillula* encompasses approximately 40 species, including *L. duriaei, L. cylindrosposa*, *L. guilanensis*, *L. saxaouli* and *L. taurica* (Braun and Cook 2012), which are among the best‐characterised species at the molecular level. Classification within this genus has been based mainly on the morphology of asexual spores (conidia), a method that remains challenging because of its complexity and the lack of scientific consensus (Palti 1988). Molecular analyses on the basis of sequence similarities offer a promising avenue for refining this classification; however, *Leveillula* sequences in public databases are scarce compared to those of other powdery mildew fungi. For instance, the National Center for Biotechnology Information (NCBI) database includes only 491 sequences for *Leveillula* spp., in contrast to 100,057 sequences for *Blumeria* spp. https://www.ncbi.nlm.nih.gov/, accessed in June 2025). Notably, 71.5% of the *Leveillula* sequences correspond to conserved regions, such as nuclear ribosomal genes (rDNA) or the internal transcribed spacer (ITS) regions.

Despite the limited availability of *Leveillula* sequences in public databases, phylogenetic analyses of the *Erysiphaceae* family using 5.8S, 18S and 28S rDNA sequences reveal that all *Leveillula* species form a monophyletic group. Their closer relatives are the species' sister group of the genus *Phyllactinia*. Together with *Pleochaeta* and *Queirozia*, these four genera form the tribe *Phyllactinieae*, a monophyletic group of hemi‐endophytic fungi, unlike all the other *Erysiphaceae*, which are ectophytic (Shirouzu et al. 2020; Takamatsu 2004; Takamatsu et al. 2016; Vaghefi et al. 2022). A parsimonious evolutionary scenario suggests that the hemi‐endophytic lifestyle emerged once in their common ancestor. Although *Phyllactinia* species are predominantly recorded in the Americas, *Leveillula* species are prevalent in Asia and the Mediterranean region. Accordingly, Takamatsu (2013) proposed that *Leveillula* and *Phyllactinia* diverged during the Paleogene Period (66 to 23 million years ago), a time when the opening of the North Atlantic Ocean separated the Eurasian and North American plates, limiting migration and likely facilitating speciation. However, Bradshaw et al. (2025) recently reconstructed the phylogeny of species of these genera using concatenated sequences of ITS, calmodulin, β‐tubulin and the second largest subunit of RNA polymerase. Their results placed the studied *Leveillula* specimens within the large *Phyllactinia* clade. On the basis of this, the authors proposed reducing the genus *Leveillula* to synonymy with the genus *Phyllactinia*, supported by the shared production of dimorphic conidia in both genera.

**FIGURE 1:**
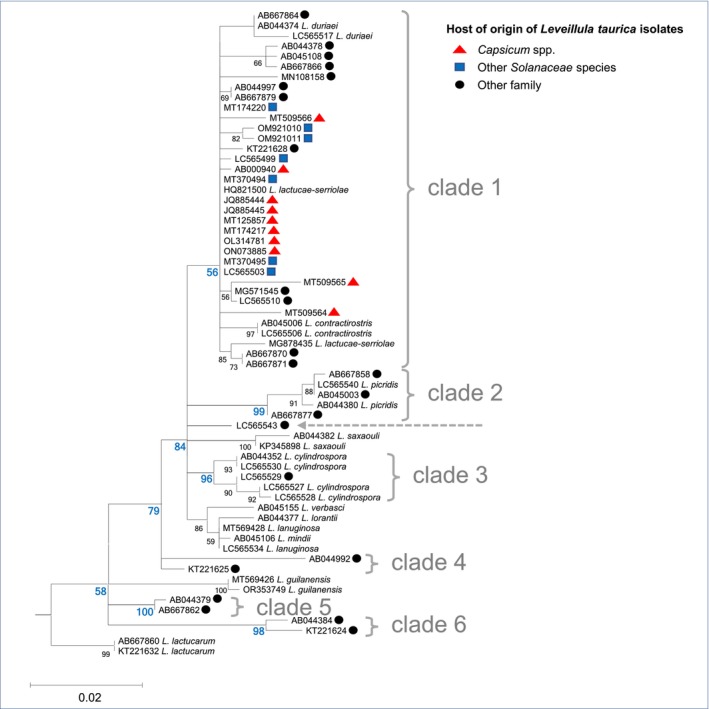
Phylogenetic tree of *Leveillula* spp. on the basis of a 575‐nucleotide‐long alignment of 63 rDNA internal transcribed spacer (ITS) sequences. NCBI accession numbers, which are listed in Table S1, and *Leveillula* species names (except for members of *L. taurica*) are indicated. Sequences obtained from *Leveillula* collected on *Capsicum* or other *Solanaceae* species are indicated by red triangles and blue rectangles, respectively. Two isolates of *Phyllactinia guttata*, the closest relative to genus *Leveillula*, were used to root the tree. Sequences were aligned with the Muscle software implemented in the MEGA 11 software (Tamura et al. 2021) and refined by eye. The tree was constructed using the maximum‐likelihood method and the partial deletion option with MEGA 11. The Kimura 2‐parameter substitution model (Kimura 1980) with a gamma distribution of rates across sites was selected. The consensus tree is shown, with bootstrap values > 50% (on the basis of 1000 replicates) indicated on the left of nodes (Felsenstein 1985). Bootstrap values supporting the six paraphyletic clades containing *L. taurica* isolates are in blue. The dotted grey arrow points to a *L. taurica* isolate that does not belong to these clades. The scale bar indicates evolutionary distances in nucleotide substitutions per site.

Building upon public sequences, we expanded the phylogenetic analysis of *Leveillula* species by using ITS sequences (Table S1). Our findings confirm that *L. taurica* does not form a monophyletic group within the genus *Leveillula*. Instead, it is distributed across at least six paraphyletic clades (Figure 1). This observation aligns with earlier studies by Khodaparast et al. (2001) and Palti (1974), suggesting that *L. taurica* represents a species complex rather than a single, well‐defined species. Notably, three of these clades (clades 1 to 3, Figure 1) include other *Leveillula* species, underlining the complexity of species delineation based solely on conidial morphology and highlighting the need for molecular phylogenetic studies to further improve taxonomy. However, all *Leveillula* specimens collected from *Solanaceae* hosts were identified as *L. taurica* and clustered within clade 1, suggesting a common evolutionary origin. Further studies incorporating DNA sequences of additional genes and broader sampling are necessary to achieve a more comprehensive understanding of the evolutionary relationships within this species complex.

## 3 Genomics of Hemi‐Endophytic *Erysiphaceae*

Compared to well‐studied plant‐pathogenic fungi such as *Fusarium* and *Magnaporthe*, the genomics and genetic basis of pathogenicity in powdery mildew remain poorly understood. To date, only a few *Erysiphaceae* genomes have been sequenced, including that of *Pseudoidium neolycopersici* (synonym: *Oidium neolycopersici*), a pathogen of tomato (Wu et al. 2018). Powdery mildew genomes are unusually large, ranging from 28 to 222 Mb, largely because of their high content of transposable elements (TEs), which can comprise 9% to 85% of the genome. Gene content is relatively low, typically between ~6000 and ~8500 genes, reflecting extensive gene loss, especially in genes related to primary and secondary metabolism, likely an adaptation to their obligate biotrophic lifestyle (Spanu et al. 2010). Between 70 and over 800 candidate secreted effector proteins (CSEPs) could delay or inhibit host immune responses. Some TEs are associated with effector genes, suggesting a possible role in the evolution of pathogenicity (Barsoum et al. 2019). In addition, sexual recombination between divergent *B. tritici* *formae speciales* has led to the emergence of new strains capable of adapting to new crops (Menardo et al. 2016).

Regarding *L. taurica*, advancements in genomics have further enriched our understanding. Kusch et al. (2020) recently delivered the first full‐genome sequence of this fungus, derived from an isolate collected on *Capsicum annuum* in Hungary (herbarium deposit: MYC‐006405). The draft genome spans 192.7 Mb, organised in 23,599 scaffolds, with TEs constituting 78.8% of the genome, the third‐highest percentage recorded among powdery mildew fungi. This abundance of TEs suggests significant adaptability to biotic and abiotic stresses (Kusch et al. 2023).

Kusch et al. (2020) also generated a transcriptome from an Australian isolate collected on *C. annuum* (herbarium deposit: BRIP 70887), yielding a genome with a BUSCO (Benchmarking Universal Single‐Copy Orthologs) score of 86%. Although this additional draft genome constitutes an important step in understanding the genetic structure of *L. taurica*, it remains highly fragmented and is based on only two isolates collected from the same host species.

These *Leveillula* genomic resources, along with the complete genome of members of genera *Phyllactinia* and *Pleochaeta* (Kusch et al. 2022), mark a significant milestone in unravelling the genetic structure and evolutionary dynamics of the hemi‐endophytic *Erysiphaceae*. However, further genomic studies encompassing a broader range of isolates collected on diverse host species are needed to provide deeper insights into their biology and pathogenicity.

## 4 Epidemiology of *L. taurica*

### 4.1 Geographical Distribution

To analyse the global distribution of *L. taurica* over time, we compiled a comprehensive database summarising information from scientific literature, herbarium records, GenBank entries, technical reports and online databases (Cussonneau et al. 2025). This database spans from 1851 to 2023 and records the presence of *L. taurica* along with associated data, such as geographic location, host plant species, DNA sequence data, presence of the sexual form and microscopic observations (e.g., number of asci and ascospores). Here, an “occurrence” is defined as a report of *L. taurica* on a specific host species in a given year and country. To reduce redundancy and sampling bias, the 342 reports of *L. taurica* on *Astragalus lentiginosus* from the Great Basin Desert of the Sierra Nevada (USA) in 2014 were consolidated into a single occurrence (Harrison et al. 2018). After this curation step, 924 occurrences out of the 1468 records compiled in our database were retained for analysis.

**FIGURE 2:**
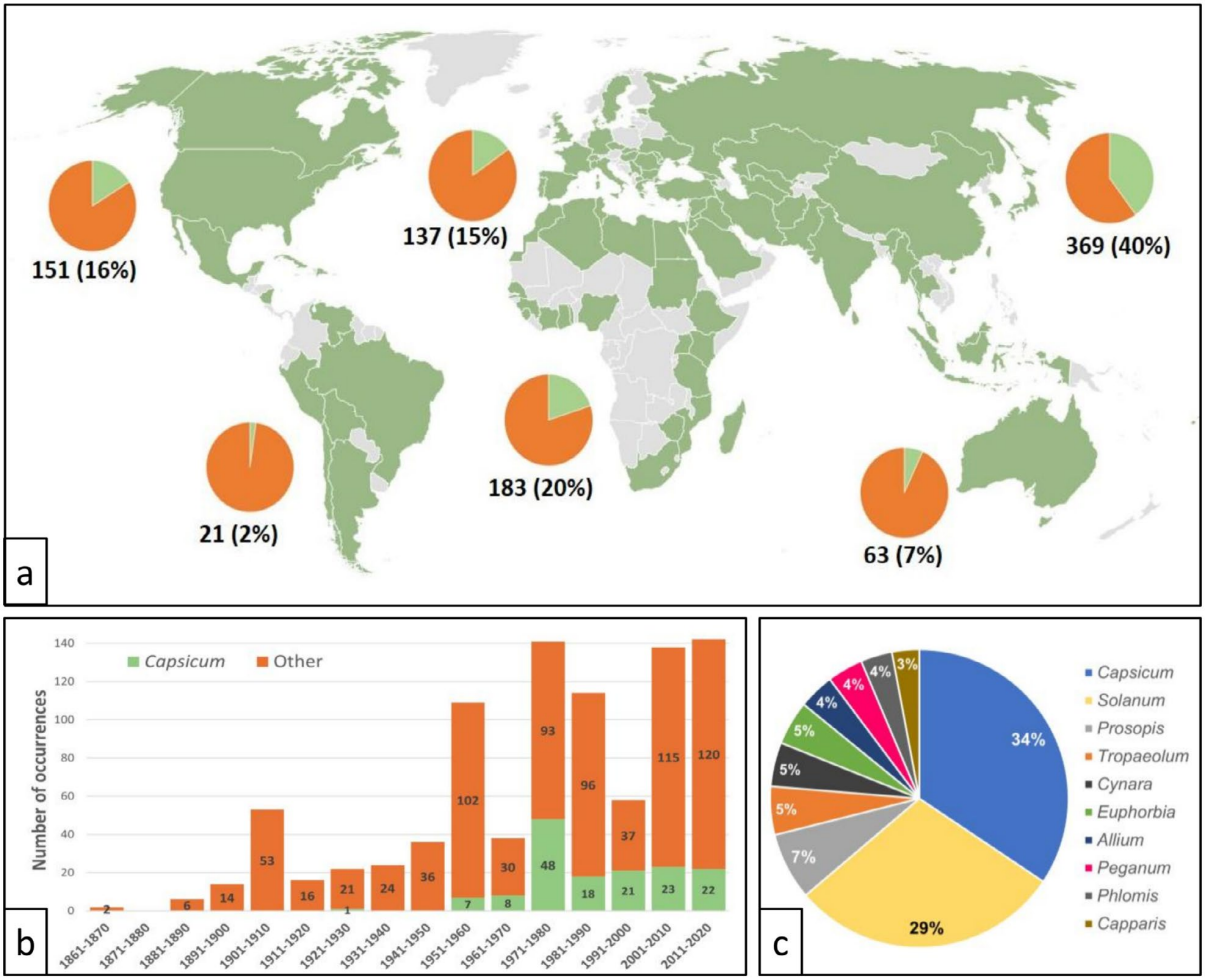
Geographical distribution (a), literature occurrence during the 1861–2020 period (b), and host range (c) of *Leveillula taurica*. The graphs are based on 924 occurrences of *L. taurica* described on a specific host species in a given year and country, selected from the occurrence database (Cussonneau et al. 2025). (a) The worldwide presence of *L. taurica*, by country from 1861 to 2020, is indicated in green. Pie charts, by continent, represent the proportion of occurrences on the genus *Capsicum* (green) and on the other plant genera (orange). Numbers (and percentages) below the pie charts indicate the number (and percentage) of occurrences in the continent. (b) Evolution of the worldwide number of occurrences per decade with the proportion of occurrences on the genus *Capsicum* (green) among the other genera (orange). (c) Pie chart representing the proportion of occurrences of *L. taurica* in the top 10 host genera, representing 48% of the 924 total occurrences, of which occurrences in *Capsicum* spp. represent 16%.

Our data reveal that *L. taurica* is widespread throughout the world, with the highest prevalence in Central Asia (40% of occurrences) (Figure 2a). Notably, its distribution overlaps substantially with major pepper cultivation areas worldwide (CABI, September 2024).

The number of reported *L. taurica* occurrences per decade, regardless of host plants, has risen sharply over time (Figure 2b). Although this trend may indicate an increase in disease incidence, it could also reflect enhanced surveillance and a larger volume of published research. Intriguingly, occurrences have roughly doubled on average in the past two decades compared to the period between 1981 and 2000.

The broad global distribution of *L. taurica* may be attributed to its adaptability to diverse agroecosystems. Alternating day and night temperatures promote *L. taurica* infections, as evidenced by our unpublished records of severe outbreaks in the Jordan Valley and Moroccan greenhouses, where daily temperatures fluctuate between 10°C and 52°C. *L. taurica* can also survive in extreme environments, including arid regions like the Great Basin Desert where diurnal temperatures substantially fluctuate (Harrison et al. 2018). Additionally, in vitro studies show that *L. taurica* can survive for 2 months at −10°C within infected pepper leaves (Cerkauskas et al. 2011). Takamatsu (2004) hypothesised that its hemi‐endophytic lifestyle may enhance its resilience to dry conditions, facilitating its adaptation to xerophyte habitats.

### 4.2 Host Range

Powdery mildew fungi are often regarded as having a narrow host range (Bardin and Gullino 2020) and generally need only one host species to complete their life cycle (Barsoum et al. 2019). However, *L. taurica* is known to have a broad host range. According to our database (Cussonneau et al. 2025), *L. taurica* can infect members of 192 plant genera across 60 families. The 10 most commonly infected genera account for 48% of occurrences, with *Capsicum* being the most frequently reported host genus accounting for 16% of the 924 occurrences (Figure 2a,c) (e.g., Clerk and Ayesu‐Offei 1967; García‐Gaytán et al. 2016; Lefebvre et al. 2003). In addition, the frequency of *L. taurica* on *Capsicum* has increased over time, peaking at 43% in the decade 1971 to 1980 (Figure 2b).

Other major hosts of *L. taurica* include members of the genus *Solanum*, such as tomato and eggplant, which together account for 14% (e.g., Bubici and Cirulli 2008; Lin et al. 2022; Mahrishi et al. 1979; Thomson and Jones 1981), followed by the genus *Prosopis* (mesquite) (3%) (e.g., Abkhoo 2015; Little 2006) and the monocotyledonous *Allium* (2%) (e.g., Du Toit et al. 2004; Vakalounakis 2016). The fungus also infects other economically important crops such as genera *Cynara* (artichoke) (Aydoğdu et al. 2021; Correll et al. 1987; Palti 1988), *Capparis* (caper) (Gupta and Bhardwaj 1998; Sepahvand et al. 2021), *Cucumis* (Vakalounakis et al. 1994; Beltrán‐Peña et al. 2018) and *Gossypium* (cotton) (Nour 1957), as well as ornamental genera, such as *Tropaeolum* (nasturtium) (Xiao et al. 2021), *Euphorbia* (poinsettia) (Khodaparast et al. 2001; Kiss et al. 2020), *Peganum* (harmel) (Ahmed and Abdel‐Azeem 2015), and *Phlomis* (Jerusalem sage) (several herbarium deposits on https://www.mycoportal.org; listed in the *L. taurica* occurrence database; Cussonneau et al. 2025).

Additionally, it has been reported on herbaceous weeds, including *Cirsium arvense* (creeping thistle), *Sonchus asper* (prickly sow‐thistle), *Convolvulus arvensis* (field bindweed), and *Xanthium* spp. (cocklebur) (Correll et al. 1987; Gupta and Bhardwaj 1998; Javadi et al. 2006), as well as perennial tree species, such as *Combretum glutinosum* in Guinea (Amano 1986) and *Moringa oleifera* in Ethiopia and India (Bartíková et al. 2020; Ullasa et al. 1981).

Historically, the genera *Phyllactinia* and *Leveillula* were distinguished by their host preference: *Phyllactinia* predominantly infects woody plants, whereas *Leveillula* primarily infects herbaceous species (Saenz and Taylor 1999). However, the occurrence of *L. taurica* on trees suggests that it was originally adapted to woody hosts before shifting partially to herbaceous hosts (Takamatsu 2013). This evolutionary shift may explain its broad host range (Palti 1988). With the separation between the genera *Leveillula* and *Phyllactinia* being one of the most recent within the *Erysiphaceae* family (Takamatsu 2013), the extensive host range and global distribution of *Leveillula* spp. indicate recent and rapid expansion, as well as a remarkable evolutionary potential.

Cross‐inoculation studies have shown that *L. taurica* isolates collected from one host genus can infect both *Solanaceae* and non‐*Solanaceae* species. Isolates from pepper, tomato, eggplant, cucumber and artichoke have successfully infected a wide range of hosts across diverse plant families (Ayesu‐Offei 1989; Correll et al. 1987; De Souza and Café‐Filho 2003; Molot et al. 1980). In rare cases, reduced infectivity on certain hosts has been observed, which may suggest host specificity or the existence of *formae speciales* (host‐adapted forms). However, such host limitations could also result from inadequate environmental conditions or poor inoculum quality (De Souza and Café‐Filho 2003). Overall, the broad host range of *L. taurica* poses epidemiological challenges. Weed hosts such as *Nicandra physaloides*, *Chenopodium ambrosioides* and *Sonchus oleraceus* , as well as winter crops like onion, may act as reservoirs, enabling the pathogen to overwinter and initiate primary infections (Correll et al. 1987; De Souza and Café‐Filho 2003).

### 4.3 Life Cycle

**FIGURE 3:**
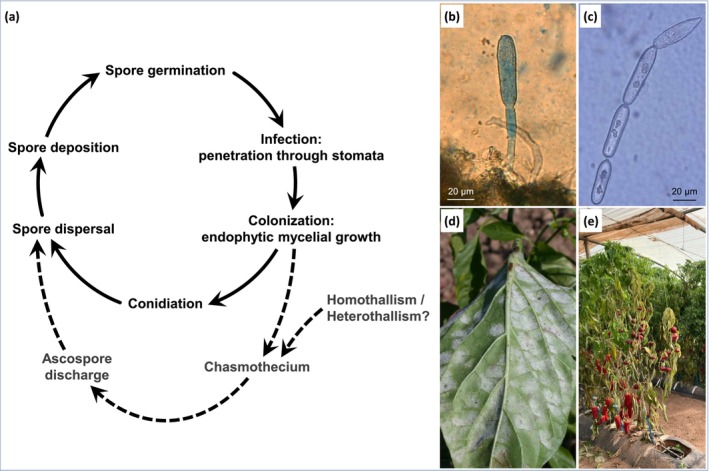
Life cycle of *Leveillula taurica*. (a) Solid arrows indicate the asexual pathway and dotted arrows indicate the sexual pathway, which has never been observed on pepper and whose epidemiological importance is unclear. (b) Production of conidia on the plant. (c) Morphology of asexual spores with lanceolate primary conidia and cylindrical secondary conidia. (d) Sporulation on the abaxial surface of a pepper leaf. (e) Colonisation of pepper by the fungus, showing leaf shedding.

Figure 3 illustrates the overall life cycle of *L. taurica*. Like most members of the *Erysiphaceae*, the fungus exhibits an asexual phase characterised by the prolific production of air‐dispersible conidia. It also has a hypothetical sexual phase, although its epidemiological importance remains uncertain.

*Primary infection and environmental conditions *—Infection begins when conidia land on a host leaf. Optimal conditions for early development range from 25°C to 35°C with 75% to 100% relative humidity (Elad et al. 2007; Nour 1958; Palti 1988). Despite its affinity for high humidity, *L. taurica* conidia can germinate at relative humidity as low as 0% to 30% (Nour 1958) and remain viable for at least 24 h on desiccated leaves (10% to 15% relative humidity) maintained at 21°C (Bardin et al. 2007). The ascospore infection process is not well documented for the fungi belonging to the *Erysiphaceae* family, particularly for *L. taurica*. However, it is widely assumed to closely resemble the conidial infection process (Jarvis et al. 2002).

*Host penetration and mycelial growth *—Upon germination, *L. taurica* produces adhesion bodies specialised for attachment to the host (Zheng, Nonomura, Bóka, et al. 2013), unlike most *Erysiphaceae* species that form appressoria for direct penetration into host cells. The mycelial hyphae then grow toward the stomata, typically more abundant on the abaxial leaf surface, and penetrate through the stomata into the substomatal cavity to establish an endophytic infection (Takamatsu 2004). This contrasts with most powdery mildews, which are ectophytic and develop externally on the adaxial leaf surface. *L. taurica* forms haustoria within host cells, siphoning water and nutrients from the cytoplasm (Glawe 2008). Among the *Erysiphaceae*, only *Leveillula*, *Phyllactinia*, *Queirozia* and *Pleochaeta* are hemi‐endophytic, meaning their mycelium grows both inside and outside host tissues, with haustoria developing inside mesophyll cells (Kunoh et al. 1981; Vaghefi et al. 2022; Zheng, Nonomura, Bóka, et al. 2013).

*Asexual reproduction and conidia dispersal *—After internal colonisation of the plant by the mycelium, conidiophores emerge from the stomata, either singly or in clusters of 2 to 5, reaching 200 to 500 μm in length on pepper. *L. taurica* conidiophores produce lanceolate conidia with a pointed tip and truncated base, and nearly rounded conidia with two truncated ends, typically measuring 30–50 × 10–20 μm (Palti 1988; Figure 3). Environmental factors such as relative humidity, temperature and wind probably play an essential role in the release and dispersal of *L. taurica* spores, as is the case for other agents responsible for powdery mildew (Qin et al. 2023; Willocquet and Clerjeau 1998; Willocquet et al. 1998). Although little is known about the efficiency of spore dispersal by wind and their survival time during airborne transport, wind is probably the main vector causing secondary infections on the plant throughout the growing season. After being deposited on leaves, the spores germinate and initiate an infection cycle.

*Sexual reproduction and overwintering strategy *—Chasmothecia (synonym: cleistothecia), the sexual structures of *Erysiphaceae* that enclose ascospores, have been observed on several host species, such as *Cynara cardunculus* , *Astragalus* spp., *Prosopis* spp. and *Ficus* spp. (Little 2006; Salari et al. 2011). In *L. taurica*, chasmothecia form within dense mycelium, typically on the abaxial leaf surface, and darken with age. They usually contain more than 20 asci (60–120 × 15–45 μm), each bearing one to four (mostly two) ascospores, although the size of ascospores varies significantly (22–54 × 12–35 μm) (Eliade 1973; Palti 1988; Sepahvand et al. 2021). The factors triggering sexual reproduction remain unclear, as does the reason for its occurrence on specific hosts. Notably, no *L. taurica* chasmothecia have been reported on pepper.

Sexual reproduction in *Erysiphaceae* can be heterothallic or homothallic, depending on the species (Brewer et al. 2011), but the reproductive mode of *L. taurica* remains unknown. As an obligate biotroph, *L. taurica* depends on living host tissues to complete its life cycle. Its sexual stage likely serves as a dormant phase during intercropping periods when hosts are scarce. Notably, because no chasmothecia have been observed on *Capsicum* spp., *L. taurica* may persist on alternative hosts, including non‐agricultural vegetation such as *Prosopis* spp., to endure unfavourable conditions (Little 2006; Palti 1988; Soylu et al. 2021).

## 5 Crop Management Practices for Controlling *L. taurica*

Control measures for reducing *L. taurica* outbreaks rely on integrated management that could combine regular crop monitoring, cultural practices, biocontrol agents, chemical treatments, and the use of resistant plant varieties.

*Crop monitoring *—Early detection is critical for controlling *L. taurica* and limiting its spread. Ideally, monitoring should involve detecting airborne spores before symptoms appear on plants (Soubeyrand et al. 2024). However, to date, this has not yet been achieved for *L. taurica*, and routine monitoring still relies on visual crop inspection. Advanced technologies, such as robotic systems using multispectral imaging or real‐time PCR assays for quantifying *L. taurica* biomass in host leaves, offer promising alternatives for improving early detection (Schor et al. 2017; Zheng, Nonomura, Bóka, et al. 2013).

*Cultural practices *—Prophylactic measures help prevent *L. taurica* infection. Given the pathogen's ability to infect multiple crops, including cucumber, tomato, pepper and eggplant, these hosts should be spatially separated to reduce cross‐infection risks (Palti 1988). Weeds that serve as reservoirs for *L. taurica* should also be removed before and during the growing season to limit the production of primary inoculum.

Nutrient management influences disease incidence. Excessive nitrogen fertilisation promotes *L. taurica* infections in tomato, so fertiliser applications should be carefully regulated to minimise epidemics (Palti 1988). Because humidity plays a key role in the disease cycle, adopting drip irrigation instead of furrow irrigation helps regulate moisture levels and reduce disease severity (Salas et al. 2015).

*Biocontrol agents *—Several biocontrol agents have been tested for controlling *L. taurica* on pepper. Spraying leaves with the mycoparasite *Ampelomyces quisqualis* (strain AQ10) limits fungal sporulation in the early stages of infection under greenhouse and field conditions (Arena et al. 2017). Similarly, *Trichoderma harzianum* (strain T39) has shown effectiveness in reducing the severity of *L. taurica* infections by inhibiting fungal growth on pepper (Elad 2000).

Additionally, natural plant extracts have demonstrated antifungal activity against *L. taurica*. Applications of tea tree oil (Timorex Gold) provide up to 80% protection in pepper plants (Arici and Özkaya 2022). Extracts from bulbs of *Allium sativum* (garlic) or *Allium cepa* (onion) or from *Azadirachta indica* (neem) have inhibited *L. taurica* conidial germination under laboratory conditions, suggesting their potential use in the greenhouse and in the field (Sudha and Lakshmanan 2007). However, as many biocontrol agents target spore germination, they must be applied at specific stages of the pathogen's life cycle and they do not provide complete disease control.

*Chemical treatments *—The management of *L. taurica* epidemics still relies heavily on chemical control methods. These include applications of inorganic compounds, such as sulphur‐based fungicides, commonly used in organic farming, and synthetic fungicides, such as demethylation inhibitors (DMIs) fungicides (e.g., tebuconazole) and strobilurin‐based fungicides (quinone outside inhibitor fungicides, QoI) (e.g., trifloxystrobin and azoxystrobin), which have demonstrated efficacy (Arici and Özkaya 2022; Cerkauskas et al. 2011; Gázquez et al. 2011; Smith et al. 1999). A list of treatments identified in the literature is available in Table S2.

Inorganic compounds such as monopotassium phosphate (KH_2_PO_4_) and potassium bicarbonate (KHCO_3_) have proven effective against *L. taurica*. Foliar application of a 1% (wt/vol) KH_2_PO_4_ fertiliser solution reduced fungal growth, sporulation and conidial production in greenhouse‐grown peppers. This treatment, which acts both locally and systemically, matched the efficacy of conventional systemic fungicides without causing phytotoxicity or yield loss (Reuveni et al. 1998). KHCO_3_ inhibits spore germination and fungal development by increasing leaf‐surface pH and has also been shown to lower disease severity under both greenhouse and field conditions (Cerkauskas et al. 2011). Both treatments are considered environmentally friendly, with low toxicity and no harmful residues, making them suitable for organic and integrated disease management programmes.

QoI fungicides, which target fungal cytochrome b, are commonly used against pepper powdery mildew. However, some *L. taurica* isolates harbour mutations in genes involved in cytochrome b formation, rendering them highly resistant to these fungicides (Mosquera et al. 2019). Beyond resistance issues that compromise efficacy, the use of synthetic fungicides raises concerns regarding environmental and human health issues.

Ultimately, a sustainable approach to *L. taurica* control in pepper crops requires combining varietal resistance, prophylactic measures, optimised agricultural practices and biocontrol methods. This integrated strategy can help reduce dependency on synthetic fungicides while maintaining effective disease management.

## 6 Pepper as a Major Host for *L. taurica*

### 6.1 A Widely Cultivated Crop

The genus *Capsicum* comprises 43 species (Barboza et al. 2022), 5 of which are cultivated: *C. annuum* , *C. chinense* , *C. frutescens* , *C. baccatum* and *C. pubescens* . In terms of surface acreage, *Capsicum* ranks as the second most widely grown fruit and vegetable in the world after tomato. It is cultivated in 142 countries across all continents except Antarctica, predominantly in tropical and subtropical regions. In 2023, the global pepper production reached 61.6 million tonnes, covering almost 4.7 million hectares (FAO 2023). Peppers are grown in open fields, plastic tunnels and greenhouses for fresh consumption, drying or processing. However, production faces significant threats from various pests and pathogens, including *L. taurica*. The latter causes significant yield losses in *Capsicum*, with which it shares an extensive geographical distribution.

### 6.2 Damages Caused by *L. taurica*

Losses caused by *L. taurica* are generally substantial, varying with the timing of infection, crop management, environmental conditions and pepper variety. The disease impacts both greenhouse and open‐field productions. In untreated open fields, yield losses have reached 50% in India (Bademiyya and Ashtaputre 2019; Palti 1988) and 50% to 60% in the United States (Smith et al. 1999). In hydroponic greenhouses, *L. taurica* caused 50% to 75% defoliation in the United States (Damicone and Sutherland 1999). These global records, spanning different years and cultivation systems, highlight the critical need for effective disease management to secure pepper production.

### 6.3 Host Resistance

Plants deploy multiple defence mechanisms against powdery mildew pathogens. These include preformed physical and chemical barriers, pattern‐triggered immunity (PTI) activated upon recognition of pathogen‐associated molecular patterns (PAMPs), effector‐triggered immunity (ETI) often mediated by resistance (*R*) genes and resistance by loss of susceptibility (for review, see Lefebvre et al. 2020). Major *R* genes to powdery mildews are more commonly observed in monocotyledonous species than in dicotyledonous ones (Wu et al. 2018). For instance, several effector candidates AVR_MLA_ discovered in *B. graminis* f. sp. *hordei* have been shown to interact specifically with MLA resistance proteins in barley (Saur et al. 2019). In wheat, the *Pm3* locus comprises at least 17 alleles conferring race‐specific resistance to *B. graminis* f. sp. *tritici*, whereas a non‐functional variant results in susceptibility to all known isolates (Bourras et al. 2019). Besides dominant genes, recessive‐inherited resistance that corresponds to loss‐of‐function alleles of powdery susceptibility genes, such as *mlo* genes and the *ol‐2* gene, confers broad‐spectrum resistance to *B. graminis* f. sp. *hordei* in barley and *P. neolycopersici* in tomato, respectively (Bai et al. 2008; Kusch and Panstruga 2017). In addition to these well‐known resistance types, quantitative resistance, controlled by multiple loci, known as quantitative trait loci (QTLs) and each QTL having a partial effect on the host resistance, contributes significantly to a durable and broad‐spectrum defence. For instance, powdery mildew resistance QTLs have been characterised in barley against *B. graminis* f. sp. *tritici* (Muranty et al. 2008) and in grapes against *E. necator* (Possamai et al. 2021).

In *Capsicum* spp., resistance to *L. taurica* is well documented, with around 150 resistant accessions reported in the literature (Table S3). Resistance sources have been identified in cultivated species ( *C. annuum* , *C. chinense* , *C. frutescens* , *C. baccatum* and *C. pubescens* ) and wild relatives (*C. chacoense* and *C. rhomboideum*). However, most accessions have been evaluated under specific environmental and temporal conditions, limiting our understanding of their broad‐spectrum and long‐term efficacy against diverse *L. taurica* isolates.

The heritability of resistance, assessed in a few studies, ranges from 0.35 to 0.86 (Blat et al. 2005a, 2005b, 2006; Daubèze et al. 1995; Lefebvre et al. 2003). Heritability is defined as the proportion of observed phenotypic variation of a trait that is attributable to genetic variation within a given population and environment. A heritability of 0 indicates that all observed variation is due to environmental factors, whereas a value of 1 means full genetic determination. In this context, a heritability of 0.35 implies moderate genetic influence, whereas values near 0.86 reflect strong genetic control. Traits with high heritability are generally easier and more efficient to improve through selection.

Despite extensive documentation on resistance sources, only a few studies have identified resistance loci conferring resistance to *L. taurica* (Table 1). The genetic determinism varies across pepper germplasm, ranging from monogenic inheritance, either dominant or recessive, to a polygenic control involving additive and epistatic effects (Blat et al. 2005a, 2005b, 2006; Lefebvre et al. 2003).

**TABLE 1:** Identified loci in pepper determining resistance to *Leveillula taurica*.

Genetic determinism of the resistance	Name or type of locus	Carrier chromosome(s)	Resistant accession(s)	Species	Publication(s)
Dominant monogenic	—	Chr. P4	MGR13FO1‐2281/1WH, registered cultivar derived from an unknown resistant accession	NA	Paran et al. 2014 (Patent US 8642845 B2)
Dominant monogenic	*PMR1*	Chr. P4	VK515 R and PM Singang	*Capsicum annuum*	Jo et al. 2017
Recessive monogenic (loss of a susceptibility factor)	*CaMlo2*	Chr. P5	VIGS of the gene *CaMlo2* in cultivar A	NA	Zheng, Nonomura, Appiano, et al. 2013
Polygenic	5 QTLs (with additive and epistatic effects)	Chr. P5, P6 (major effect), P9, P10 and P12	H3 (syn. PM00807)	*C. annuum*	Lefebvre et al. 2003
Polygenic	2 major‐effect QTLs	Chr. P4 and P6	PBC167 (syn. PI640507)	*C. annuum*	Gabor et al. 2017 (Patent US 9689045 B2); Just et al. 2020 (Patent US 0329655 A1)
Polygenic	1 QTL	Chr. P1/P8 (on the translocated segment between P1 and P8)	NCIMB_42136 registered cultivar derived from an unknown resistant accession	NA	Eggink et al. 2016 (Patent US 9351451 B2)
Polygenic	2 QTLs	Chr. P2 and P5	AR1	*C. annuum*	Manivannan et al. 2021
Polygenic	1 QTL	Chr. P6	LT17	NA	Bardol et al. 2023 (Patent WO 012325 A1)

*Note:* Pepper orthologs of *Mlo* or *PMR* genes (Acquadro et al. 2020) that have not been explicitly shown to confer resistance to *L. taurica* have not been included in Table 1.

Abbreviations: Chr., chromosome; NA, information not available; QTL, quantitative trait locus; Syn., synonym; VIGS, virus‐induced gene silencing.

#### 6.3.1 Major‐Effect Resistance Genes

Two patents reported dominant loci controlling resistance to *L. taurica* on pepper chromosome P4. One locus was introgressed into *C. annuum* line MGR13FO1–2281/1WH, registered by Hazera Genetics Ltd. in Israel (Paran et al. 2014). The other was identified in *C. annuum* accession PI640507 (also known as PBC167) from the germplasm collection of the United States Department of Agriculture (USDA) (Gabor et al. 2017).

More recently, Jo et al. (2017) identified the *PMR1* (*powdery mildew resistant 1*) locus in a 4‐Mb region of chromosome P4 in *C. annuum* accessions VK515R and PM Singang. The alignment of this region with the genomes of *C. baccatum* PI159236 and of *C. chinense* PBC81 revealed a common indel between VK515R and PI159236, suggesting *PMR1* may have resulted from an introgression event from *C. baccatum* . Within this 4‐Mb region, two genes encoding nucleotide‐binding‐site leucine‐rich‐repeat (NBS‐LRR) proteins were identified as candidates for *PMR1*, because NBS‐LRR receptors are known to be essential for pathogen detection and trigger plant defence responses, including hypersensitive reactions (McHale et al. 2006).

To further characterise resistance loci on P4, we identified sequences similar to the *PMR1* region (Jo et al. 2017) and to the two loci reported by Paran et al. (2014) and Gabor et al. (2017) in the *C. annuum* CM334 reference genome (Kim et al. 2017; GenBank assembly GCA_000512255.2). These three loci cover a 14‐Mb region at the extremity of chromosome P4. Further research is needed to decipher whether resistance is conferred by a single causal gene, different alleles of the same gene, or a cluster of linked genes.

#### 6.3.2 Resistance by Loss of Susceptibility

Resistance to powdery mildew on the basis of orthologs of the *Mildew resistance locus O* (*Mlo*), first identified in barley, arises from loss‐of‐function mutations and is predominantly recessive, a mechanism conserved across various plant genera (Kusch and Panstruga 2017). In *C. annuum* , two *Mlo* orthologs have been identified: *CaMlo1* in accession AAX31277 (Panstruga 2005) and *CaMlo2* in accession JN896629 (Kim and Hwang 2012). These genes are localised on chromosomes P11 and P5, respectively, in the reference *C. annuum* Zunla‐1 v1.0 genome (Qin et al. 2014; GenBank assembly GCA_000710875.1). Although both are *Mlo* orthologs, *CaMlo1* and *CaMlo2* share only 56% sequence identity, highlighting their divergence (Kim and Hwang 2012). Zheng, Nonomura, Appiano, et al. (2013) further identified an ortholog of *CaMlo2* in the pepper expressed sequence tag (EST) SGN‐U202700 and demonstrated that silencing *CaMlo2* in a susceptible cultivar conferred resistance to *L. taurica*.

Following this discovery, Acquadro et al. (2020) identified 16 to 18 additional *Mlo* orthologs on chromosomes P2, P3 and P8 across three *C. annuum* landraces, suggesting a diverse repertoire of *Mlo* susceptibility genes in pepper. Beyond *Mlo* genes, *PMR* (*powdery mildew resistant*) genes involved in plant cell wall reinforcement act as susceptibility genes contributing to powdery mildew resistance in *Arabidopsis thaliana* (Ellinger et al. 2013). Similarly, Acquadro et al. (2020) identified 67 to 72 *PMR* orthologs in the same three *C. annuum* landraces. However, the functional role of these *Mlo* and *PMR* orthologs in resistance to *L. taurica* remains to be experimentally validated.

#### 6.3.3 QTL‐Based Resistance

Several *Capsicum* accessions exhibit partial resistance to powdery mildew determined by QTLs. The *C. annuum* accession H3, originating from Ethiopia, has been extensively studied for its strong resistance to *L. taurica* (Daubèze et al. 1995). Genetic analyses using doubled‐haploid progeny derived from an F_1_ hybrid between H3 and the susceptible line Vania identified seven genomic regions, encompassing both additive QTLs and epistatic interactions, collectively explaining over 50% of the genetic resistance variance (Lefebvre et al. 2003). These additive QTLs are located on chromosomes P5, P6, P9, P10 and P12. Notably, the favourable alleles at QTLs on P6, P9, P10 and P12 originate from the resistant parent H3, whereas the favourable allele at the QTL on P5 comes from the susceptible parent Vania. Among these, the major‐effect QTL *Lt_6.1*, located on P6, accounts for up to 26% of the phenotypic variation. The doubled‐haploid line HV12 has demonstrated robust resistance under high infection pressure in various countries, including France, Italy, Tunisia, Israel and Brazil (Allagui et al. 1995; Blat et al. 2005b; Daubèze et al. 1995; Lefebvre et al. 2003; Marchesan et al. 2009; Shifriss et al. 1992). HV12 represents a rare case of a strong resistance source validated across diverse geographical regions and environmental conditions. The strong and robust resistance of HV12 may result from the favourable combination of quantitative trait alleles originating from both H3 and Vania, as well as beneficial epistatic interactions (Lefebvre et al. 2003). However, despite this promising resistance, the precise location of the genes and polymorphisms determining the resistance remains uncertain, with mapped loci currently spanning broad marker intervals, limiting their utility in breeding programmes.

The accession PBC167 carries two resistance alleles at QTLs located on chromosomes P4 and P6 (Gabor et al. 2017). The QTL on P6 was mapped within an interval of 6.5 cM. Although the resistance allele of the QTL on P4 is insufficient on its own in conditions of high infection pressure, its combination with the resistance allele of the QTL on P6 enhances the overall level of resistance. Comparisons between the P6 QTL identified in PBC167 and that reported in H3 (Lefebvre et al. 2003) reveal a key difference: although the H3 allele confers strong resistance, the PBC167 allele has only a minor effect. A fingerprinting study using 46 markers covering the resistance alleles of H3 and PBC167 found only 65.7% similarity between them, confirming that these alleles are distinct (Just et al. 2020). Another resistant accession, LT17, also harbours a QTL on chromosome P6, explaining less than 10% of phenotypic variance. Genome‐wide genotyping of LT17, PBC167 and H3 indicates that these lines are not genetically related (Bardol et al. 2023). However, the study does not clarify their genetic similarity at the P6 QTL, leaving open the possibility that they may share the same resistance locus.

Additional QTLs have been reported in other accessions. AR1 carries resistance alleles at QTLs *Pm2.1* and *Pm5.1*, mapped to chromosomes P2 and P5, respectively, each explaining less than 10% of phenotypic variation (Manivannan et al. 2021). The patented accession NCIMB_42136 harbours a QTL located on a translocated segment between chromosomes P1 and P8, which accounts for 56.8% of resistance variation (Eggink et al. 2016). Although progress has been made in locating resistance regions, further research is needed to pinpoint the specific causal genes.

#### 6.3.4 Physiological Resistance Mechanisms

The defence mechanisms governed by resistance genes against *L. taurica* remain largely unexplored. However, graft‐transmissible resistance to *L. taurica* has been demonstrated in the *C. annuum* accession Szentesi (Albert et al. 2017). The resistance is associated with elevated NADPH oxidase activity and increased expression of pathogenesis‐related (PR) proteins, both of which enhance plant resistance. NADPH oxidases generate reactive oxygen species (ROS) upon pathogen contact, whereas PR proteins exhibit antimicrobial properties (Vermot et al. 2021). The genetic basis of the resistance of Szentesi remains undetermined.

Overexpression of the *NPR1* (non‐expresser of pathogenesis‐related gene), a key regulator of systemic acquired resistance, has been shown to enhance disease resistance in transgenic plants (Cao et al. 1994). In transgenic pepper plants constitutively expressing *CaNPR1*, symptoms of *L. taurica* are significantly reduced (Arthikala et al. 2014).

#### 6.3.5 Comparison of Powdery Mildew Resistance Loci in Pepper and Tomato

Tomato ( *Solanum lycopersicum* ), like pepper, belongs to the family *Solanaceae* and can experience occasional outbreaks of *L. taurica*, whereas the more frequent powdery mildew disease in tomato is caused by *P. neolycopersici*. Several resistance loci against powdery mildew have been identified in cultivated tomato and its wild relatives.

The dominant *Lv* locus on chromosome T12 confers complete resistance to *L. taurica* by triggering a hypersensitive response, though the responsible gene remains unidentified (Chunwongse et al. 1997; Seifi et al. 2014). Regarding resistance to *P. neolycopersici*, five dominant loci (*Ol‐1*, *Ol‐3*, *Ol‐4, Ol‐5* and *Ol‐6*) have been mapped in two clusters on chromosome T6, and one recessive locus (*ol‐2*) on chromosome T4. Notably, loss‐of‐function mutations in the *SlMlo1* gene, underlying the *ol‐2* locus, lead to resistance to fungal penetration (Zheng et al. 2016). *SlMlo1* functions thus as a susceptibility factor facilitating powdery mildew pathogenesis, and loss‐of‐function mutations in *SlMlo1* confer recessively inherited, broad‐spectrum resistance through the formation of papillae, which are physical barriers that prevent fungal entry into host cells. In addition, several *Ol*‐QTLs contributing to partial resistance to *P. neolycopersici* have been mapped: *Ol‐qtl1* overlaps the *Ol‐1*_*Ol‐3*_*Ol‐5* cluster on T6; *Ol‐qtl2* is close to *Lv*, and *Ol‐qtl3* is distant from *Lv* on T12 (Faino et al. 2012; Seifi et al. 2014).

**FIGURE 4:**
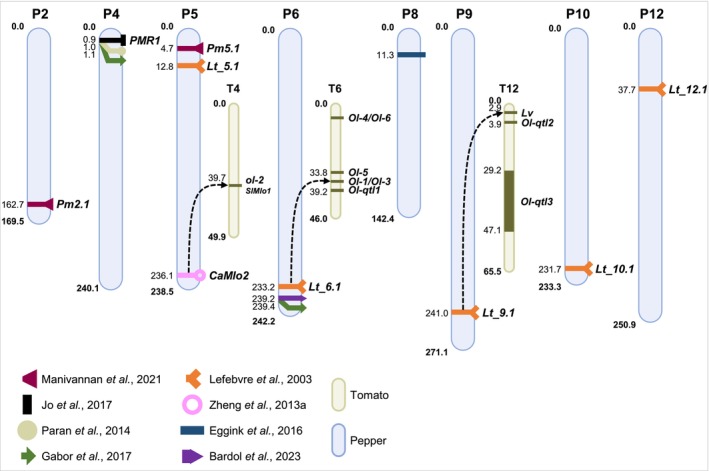
Position of *Leveillula taurica* resistance loci on the pepper genome and their syntenic position with tomato powdery mildew resistance loci. Chromosome numbers are indicated as P for pepper and T for tomato. Position in mega base pairs (Mb) on the basis of the reference genomes CM334_V1.5 for pepper and Heinz SL2.40 for tomato (Fernandez‐Pozo et al. 2015; Seifi et al. 2014; https://solgenomics.net). The arrows indicate the orthologous positions between pepper and tomato genomes, as described by Lefebvre et al. (2003) and Zheng, Nonomura, Appiano, et al. (2013). The quantitative trait locus (QTL) detected by Eggink et al. (2016) (see Table 1), initially located on the translocated segment between chromosomes P1 and P8, is illustrated here on chromosome P8.

Comparative genomic analyses reveal both conserved and species‐specific genetic bases of powdery mildew resistance in pepper and tomato (Figure 4). Synteny refers to the conservation of the order of genes or genetic loci along the chromosomes between different species because of a common ancestry, which allows for comparative mapping.

1. *SlMlo1*, located on tomato chromosome T4, is the closest tomato ortholog to the pepper gene *CaMlo2*. Loss‐of‐function mutations in *SlMlo1* confer partial resistance to *L. taurica* in both pepper and tomato (Zheng, Nonomura, Appiano, et al. 2013).
2. The *Lt_9.1* QTL on pepper chromosome P9 shows synteny with the *Lv* resistance locus on chromosome T12 (Lefebvre et al. 2003).
3. The major‐effect *Lt_6.1* QTL on chromosome P6 is syntenic with the *Ol*‐*1*/*Ol*‐*3* locus on chromosome T6 (Lefebvre et al. 2003).
4. The *PMR1* region on chromosome P4 (Jo et al. 2017) aligns with chromosome T3. However, no resistance to *L. taurica* or *P. neolycopersici* has been mapped to T3 so far.

Advances in comparative genomics may facilitate the identification of causal genes responsible for powdery mildew resistance and enhance breeding strategies in pepper (Cao et al. 2022; Wu et al. 2019). For instance, CRISPR/Cas9‐mediated impairment of *DND1* (Defence No Death 1) reduced susceptibility to *P. neolycopersici* in tomato (Li et al. 2024). To our knowledge, the role of this gene in pepper resistance to *L. taurica* remains to be studied.

## 7 Directions for Future Research

### 7.1 Advancing the Taxonomy and Biology of *L. taurica*

The taxonomy of *L. taurica*, traditionally on the basis of conidia morphology and ITS sequences, suggests that it represents a species complex. However, ITS sequences alone are insufficient for precise fungal identification because of multiple ITS copies with nucleotide variations within a single isolate, potentially leading to misclassification (Bradshaw et al. 2023). Although combining ITS with 18S, 28S and 5.8S rDNA sequences improves phylogenetic resolution (Takamatsu 2004), additional DNA barcodes like *TSR1* (20S rRNA accumulation 1), *MCM7* (minichromosomal maintenance protein 7), and *GAPDH* (glyceraldehyde‐3‐phosphate dehydrogenase) have proven more effective for distinguishing taxa within the *Erysiphaceae* family (Bradshaw and Tobin 2020; Bradshaw et al. 2022; Ellingham et al. 2019).

Whole‐genome sequencing (WGS) of multiple *L. taurica* isolates presents a promising avenue to refine its taxonomy and generate an improved reference genome (Kusch and Panstruga 2017). Long‐read‐based sequencing should enable the establishment of high‐quality genome assemblies. WGS could also elucidate key aspects of the hemi‐endophytic lifestyle of the fungus, identify genes involved in biotrophy, and clarify its reproductive state (heterothallic or homothallic). Recent studies on the mating‐type locus in *Erysiphe necator* have shed light on the role of ascospores in epidemiology (Brewer et al. 2011). Similar investigations could enhance our understanding of *L. taurica* reproduction and epidemiology. However, the non‐culturable nature of this pathogen in vitro further complicates the isolation of large, high‐quality DNA fragments, delaying the acquisition of high‐quality genomic sequences.

Expanding the collection of *L. taurica* isolates and enriching the genomic data for each isolate will refine analyses of the diversity of the species and evolutionary mechanisms driving its adaptation to different hosts or environments. Integrating refined taxonomy, life cycle insights and genomic discoveries will be essential for devising more effective and sustainable control strategies against *L. taurica*.

### 7.2 Identifying Pepper Resistance Genes

Despite reports of several resistance sources in pepper, only a few resistance genes have been identified and successfully integrated into breeding programmes. The biotrophic nature of *L. taurica* complicates both isolate maintenance and artificial inoculation, limiting research on the genetic basis of host resistance and the impact of environmental factors on resistance. Furthermore, the QTLs mapped so far span large chromosomal regions, reducing their effectiveness in marker‐assisted selection. Further fine mapping and identification of causal genes are crucial for improving breeding precision.

To date, the *CaMlo2* gene remains the only confirmed resistance gene against *L. taurica* in pepper. Although *Mlo* genes have conferred durable and broad‐spectrum resistance in barley for decades (Kusch and Panstruga 2017), their expression has shown vulnerability to stresses (Schwarzbach 2001) and a propensity to break down during experimental evolution (Kusch et al. 2023). This highlights the rapid adaptability of *L. taurica* and the need for diversified genetic resistance strategies.

A systematic evaluation of *L. taurica* resistance across diverse pepper genetic resources, environments and pathogen isolates, combined with expanded genomic data, is crucial for identifying novel resistance sources and their underlying genes. Such efforts will ultimately enhance the development of robust and sustainable resistant pepper varieties (Billaud et al. 2024).

### 7.3 Preventing Resistance Breakdown

*Leveillula taurica* poses significant research challenges because of its obligate biotroph nature, which complicates the establishment of centralised strain collections and reliable artificial inoculation protocols. Its partially endophytic lifestyle further hinders systematic DNA sampling from symptomatic hosts, as sporulation is not always observable. This results in inaccessible fungal DNA or mixed samples containing fungal, host and contaminant DNA.

Comparative field studies across diverse locations and host varieties with varying resistance levels could facilitate the early detection of isolates capable of overcoming resistance. These studies may also shed light on the selective pressures exerted by different host accessions or varieties on *L. taurica* populations, offering insights for the development of durable resistance strategies (Lefebvre et al. 2020).

By integrating genomic, ecological and phenotypic data on both *L. taurica* and *Capsicum*, researchers can better unravel the complex interplay between pathogen diversity and host resistance. This multidisciplinary approach will be essential for designing effective management strategies and ensuring long‐lasting resistance to *L. taurica*.

## Author Contributions

**Veronique Lefebvre:** conceptualization (lead), data curation (equal), formal analysis (equal), funding acquisition (lead), investigation (equal), project administration (lead), supervision (lead), validation (lead), visualization (equal), writing – original draft preparation (equal), riting – review & editing (lead). **Anne Massire:** data curation (equal), formal analysis (equal), validation (supporting), visualization (equal), writing – review & editing (equal). **Flavie Cussonneau:** formal analysis (equal), investigation (equal), visualization (equal), writing – original draft preparation (equal). **Benoit Moury:** formal analysis (equal), visualization (equal), writing – review & editing (equal). **Marc Bardin:** visualization (supporting), writing – review & editing (equal). **Sonia Elbelt:** data curation (supporting). **Crole Constant:** writing – eview & editing (supporting).

## Conflicts of Interest

The authors declare no conflicts of interest.

## Supporting information


**Table S1.** NCBI accession numbers of ITS sequences used to construct the phylogenetic tree of Figure 1.

**Table S2.** Treatments against *Leveillula taurica*.

**Table S3.** Sources of genetic resistance to *Leveillula taurica* in the genus *Capsicum*.

## Data Availability

The data that support the findings of this study are openly available in Global_Occurrence_Database_of_Leveillula_taurica at https://www.gbif.org/ accessible via the link https://doi.org/10.15468/f8bb95.
